# Supplementary material for: Light-induced stomatal opening requires phosphorylation of the C-terminal autoinhibitory domain of plasma membrane H+-ATPase
Source: Nat Commun. 2024 Feb 20;15:1195. doi: 10.1038/s41467-024-45236-9 (PMC10879506; doi:10.1038/s41467-024-45236-9)
Supplement: Supplementary file 1 — Supplementary Figure [file 41467_2024_45236_MOESM1_ESM.pdf]

Light-induced stomatal opening requires phosphorylation of the C-terminal autoinhibitory domain of plasma membrane H^+-ATPase

Saashia Fuji, Shota Yamauchi, Naoyuki Sugiyama, Takayuki Kohchi, Ryuichi Nishihama, Ken-ichiro Shimazaki, Atsushi Takemiya

Supplementary Figures 1–9

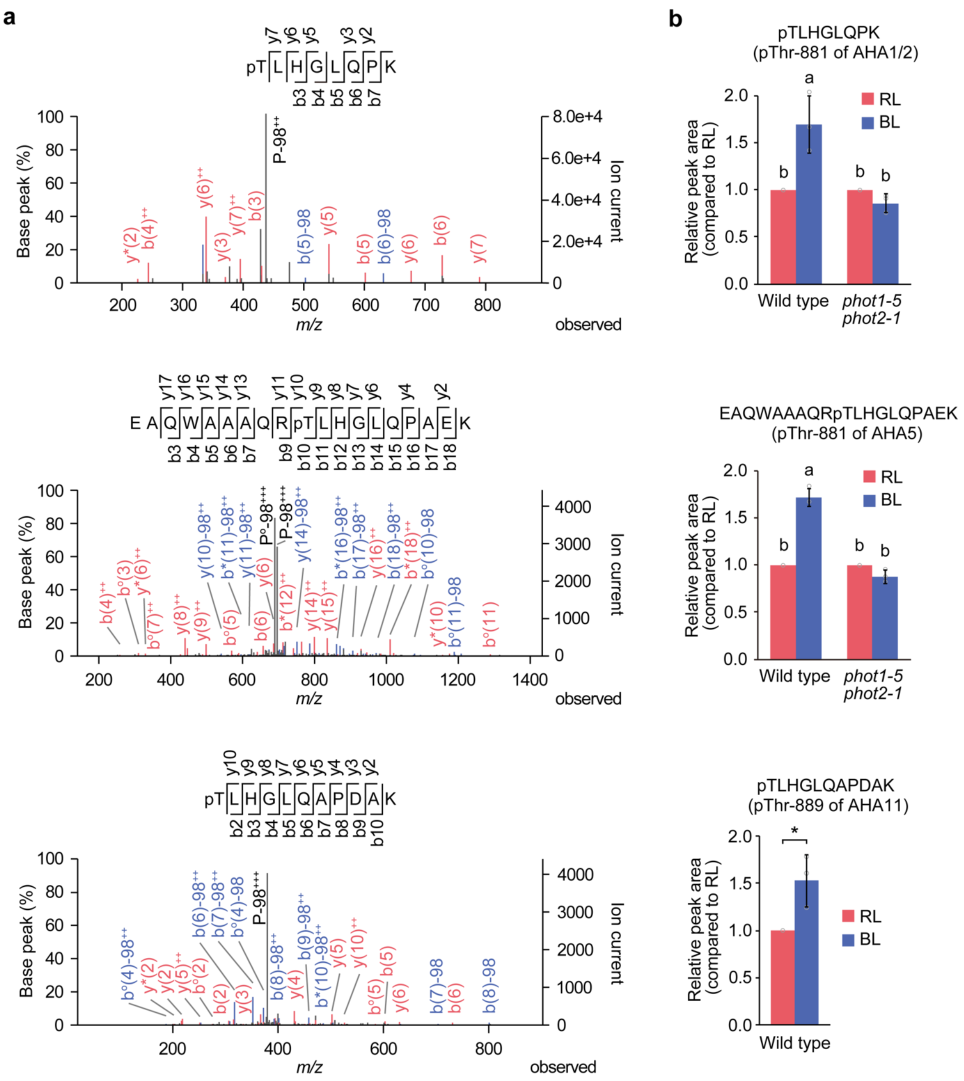

Supplementary Fig. 1 Blue light-dependent phosphorylation of AHA proteins.

a MS/MS spectra of AHA1/2, AHA5, and AHA11 phosphopeptides. The labels "-98", "*", and "o" represent neutral loss of H_3PO_4, NH_3, and H_2O from b, y, and precursor (P) ions, respectively. **b** Quantification of phosphopeptide amounts calculated from integrated peak values. The data represent means ± SD (n = 3 biologically independent samples). For AHA1/2 and AHA5, different letters indicate significant differences (One-way ANOVA with Tukey's test, $P < 0.01$). For AHA11, asterisk indicates significant differences (two-sided Student's t-test, $P < 0.05$).

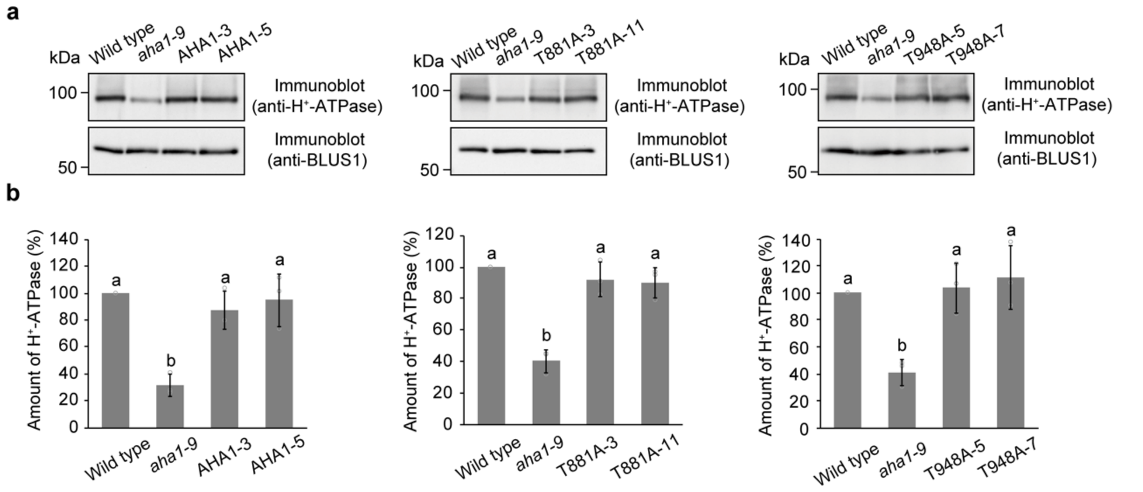

Supplementary Fig. 2 Expression of T881A and T948A variants in the *aha1-9* background.

a Immunoblot analysis using anti-H^+-ATPase antibodies. Each lane contains 4.2 µg of guard cell proteins. BLUS1 was used as the loading control. **b** Quantification of the expression level of H^+-ATPase using the ImageJ software. The expression level of H^+-ATPase is expressed as a percentage of that in the wild-type. The data represent means ± SD (n = 3 biologically independent experiments). Different letters indicate significant differences (One-way ANOVA with Tukey's test, $P < 0.01$).

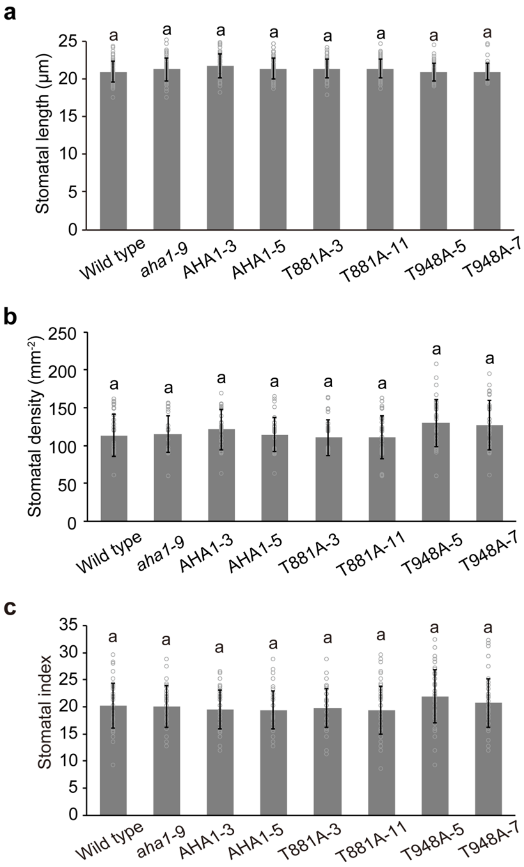

Supplementary Fig. 3 Stomatal size, density, and index in transgenic plants expressing T881A and T948A variants.

a–c Stomatal size (**a**), density (**b**), and index (**c**) in the abaxial epidermis. For **(a)** and **(c)**, the data represent means ± SD (*n* = 60 biologically independent samples). For **(b)**, the data represent means ± SD (*n* = 36 biologically independent samples). Different letters indicate significant differences (One-way ANOVA with Tukey's test, $P < 0.01$).

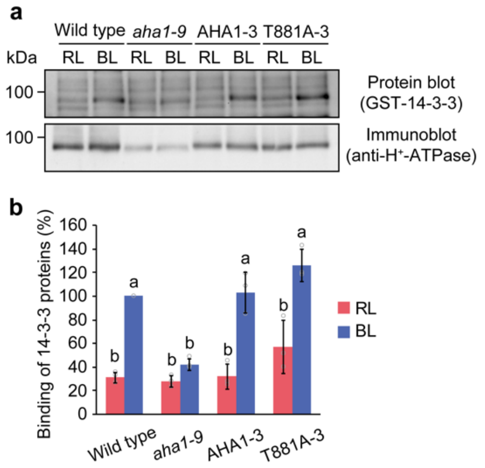

Supplementary Fig. 4 Binding of 14-3-3 proteins to H^+-ATPase.

a Guard cell protoplasts were illuminated with red light (RL: 300 μmol m^{-2} s^{-1}) for 30 min, after which a pulse of blue light (BL: 100 μmol m^{-2} s^{-1}, 30 s) was superimposed on RL. Binding of 14-3-3 proteins to H^+-ATPase was detected by protein blot analysis using GST-14-3-3 as a probe. **b** Relative binding of 14-3-3 proteins to H^+-ATPase was quantified using the ImageJ software. Each binding level was expressed as a percentage of that in the wild type under BL. The data represent means ± SD ($n = 3$ biologically independent experiments). Different letters indicate significant differences (One-way ANOVA with Tukey's test, $P < 0.01$).

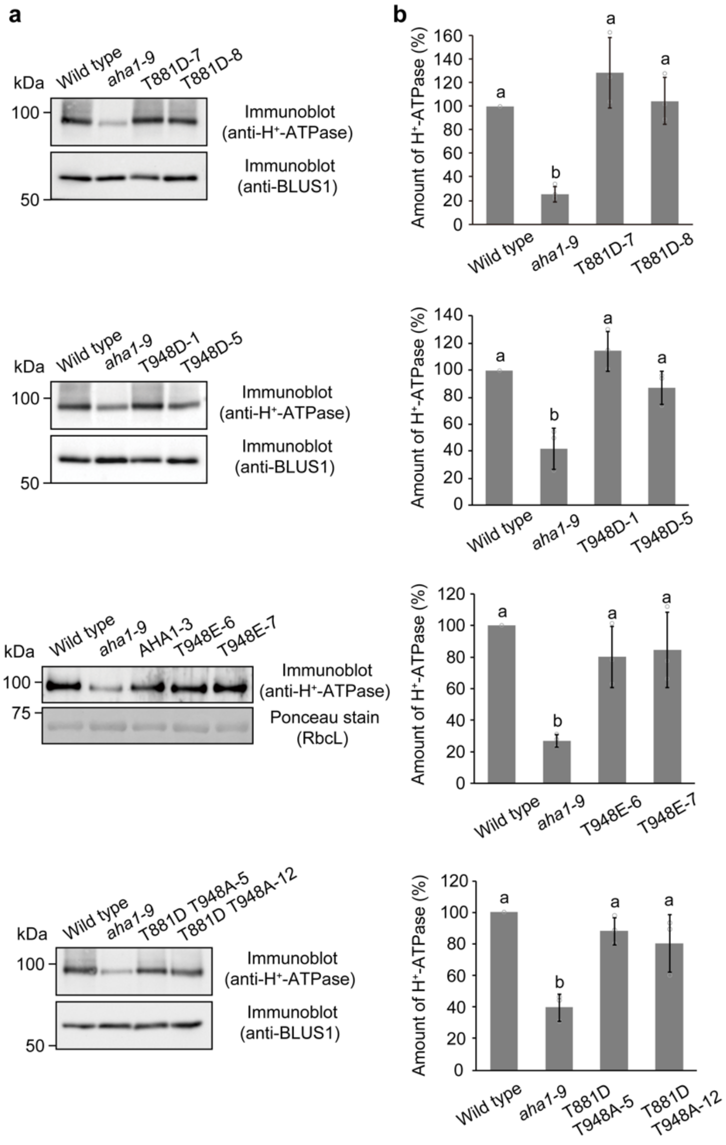

Supplementary Fig. 5 Expression of T881D, T948D, T948E, and T881A T948A variants in the *aha1-9* background.

a Immunoblot analysis using anti-H^+-ATPase antibodies. Each lane contained 4.2 µg of guard cell proteins. BLUS1 and RbcL were used as loading controls. **b** Quantification of the expression level of H^+-ATPase using the ImageJ software. The expression level of H^+-ATPase was expressed as a percentage of that in the wild-type. The data represent means ± SD (n = 3 biologically independent experiments). Different letters indicate significant differences (One-way ANOVA with Tukey's test, $P < 0.05$).

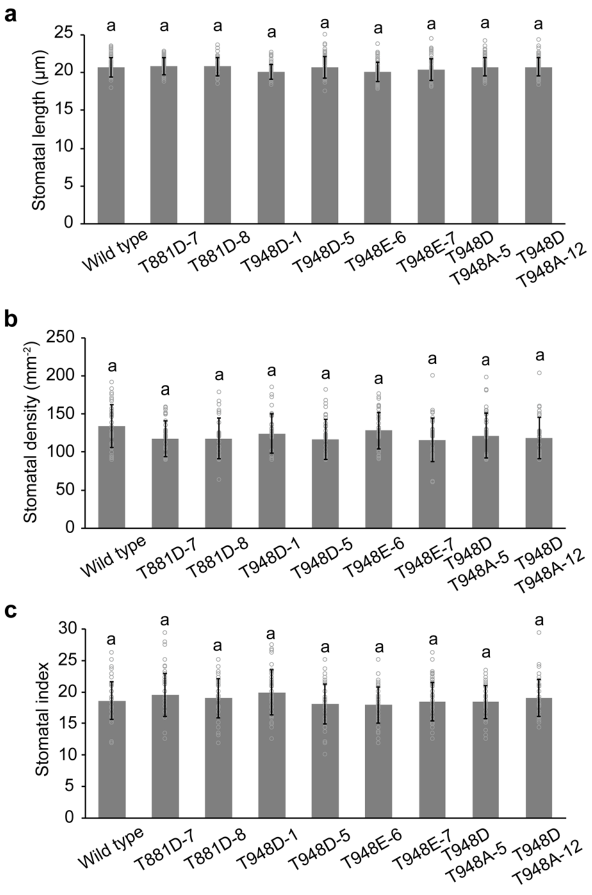

Supplementary Fig. 6 Stomatal size, density, and index in transgenic plants expressing T881D, T948D, T948E, and T881D T948A variants.

a-c Stomatal size (**a**), density (**b**), and index (**c**) in the abaxial epidermis. For **(a)** and **(c)**, the data represent means ± SD (*n* = 60 biologically independent samples). For **(b)**, the data represent means ± SD (*n* = 36 biologically independent samples). Different letters indicate significant differences (One-way ANOVA with Tukey's test, $P < 0.01$).

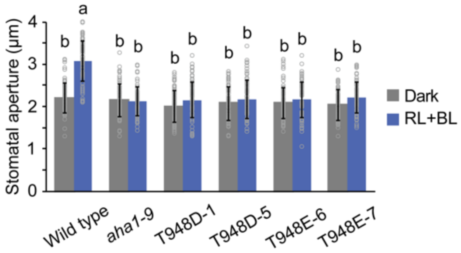

Supplementary Fig. 7 Light-dependent stomatal opening in transgenic plants expressing T948D and T948E variants.

Epidermal strips were incubated in the dark or under red light (RL: 50 μmol m^{-2} s^{-1}) with blue light (BL: 10 μmol m^{-2} s^{-1}) for 2 h. The data represent means ± SD (n = 75 stomata from three independent experiments). Different letters indicate significant differences (One-way ANOVA with Tukey's test, $P < 0.01$).

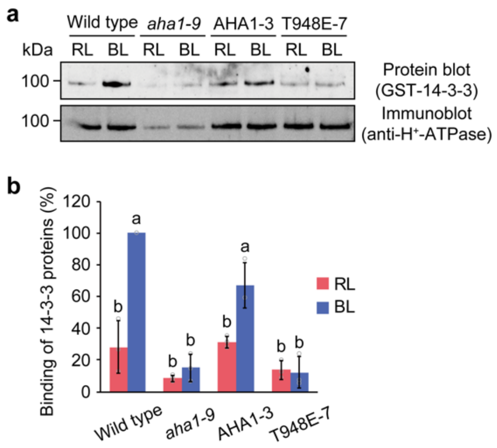

Supplementary Fig. 8 Impairment of the binding of 14-3-3 proteins to T948E variants.

a Guard cell protoplasts were illuminated with red light (RL: 600 µmol m^{-2} s^{-1}) for 30 min, after which a pulse of blue light (BL: 100 µmol m^{-2} s^{-1}, 30 s) was superimposed on RL. Binding of 14-3-3 proteins to H^+-ATPase was detected by protein blot analysis using GST-14-3-3 as a probe. **b** Relative binding of 14-3-3 proteins to H^+-ATPase was quantified using the ImageJ software. Each binding level was expressed as a percentage of that in the wild type under BL. The data represent means ± SD ($n = 3$ biologically independent experiments). Different letters indicate significant differences (One-way ANOVA with Tukey's test, $P <$ 0.01).

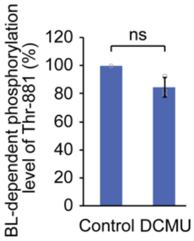

Supplementary Fig. 9 Quantification of blue light-dependent phosphorylation of Thr-881 in the presence or absence of DCMU.

Guard cell protoplasts were pre-treated with DCMU (10 μM) in the dark for 30 min, then illuminated with red light (RL: 300 μmol m^{-2} s^{-1}) for 30 min, after which a pulse of blue light (BL: 100 μmol m^{-2} s^{-1}, 30 s) was superimposed on RL. Phosphorylation levels of Thr-881 (Fig. 5c) were quantified using the ImageJ software. Blue light-dependent phosphorylation of Thr-881 was calculated by subtracting the phosphorylation levels under red light from those under blue light and expressed as a percentage relative to control. The data represent means ± SD (n = 3 biologically independent experiments). ns indicates no significant difference (two-sided Student's t-test, $P < 0.01$).
